# Excessive collagen turnover products are released during colorectal cancer progression and elevated in serum from metastatic colorectal cancer patients

**DOI:** 10.1038/srep30599

**Published:** 2016-07-28

**Authors:** S. N. Kehlet, R. Sanz-Pamplona, S. Brix, D. J. Leeming, M. A. Karsdal, V. Moreno

**Affiliations:** 1 Nordic Bioscience A/S, Herlev, Denmark; 2 Center for Biological Sequence Analysis, Department of Systems Biology, Technical University of Denmark, Denmark; 3 Unit of Biomarkers and Susceptibility, Cancer Prevention and Control Program, Catalan Institute of Oncology (ICO), IDIBELL and CIBERESP, Hospitalet de Llobregat, Barcelona, Spain; 4 Department of Clinical Sciences, Faculty of Medicine, University of Barcelona, Barcelona, Spain

## Abstract

During cancer progression, the homeostasis of the extracellular matrix becomes imbalanced with an excessive collagen remodeling by matrix metalloproteinases. As a consequence, small protein fragments of degraded collagens are released into the circulation. We have investigated the potential of protein fragments of collagen type I, III and IV as novel biomarkers for colorectal cancer. Specific fragments of degraded type I, III and IV collagen (C1M, C3M, C4M) and type III collagen formation (Pro-C3) were assessed in serum from colorectal cancer patients, subjects with adenomas and matched healthy controls using well-characterized and validated ELISAs. Serum levels of the biomarkers were significantly elevated in colorectal cancer patients compared to subjects with adenomas (C1M, Pro-C3, C3M) and controls (C1M, Pro-C3). When patients were stratified according to their tumour stage, all four biomarkers were able to differentiate stage IV metastatic patients from all other stages. Combination of all markers with age and gender in a logistic regression model discriminated between metastatic and non-metastatic patients with an AUROC of 0.80. The data suggest that the levels of these collagen remodeling biomarkers may be a measure of tumour activity and invasiveness and may provide new clinical tools for monitoring of patients with advanced stage colorectal cancer.

Colorectal cancer (CRC) is the third most common cancer and the fourth most common cause of cancer-related death worldwide, accounting for roughly 600.000 deaths per year^1^. The 5-year survival rate decreases drastically with advanced stages, being 90% for local tumours in initial stages and 12% in advanced stages with metastasis^2^. Clinical symptoms often appear at the late stages when the tumour has started to metastasize resulting in late diagnosis and lower survival rates^3^. CRC screening has resulted in a reduction in mortality^4^ but the optimal diagnostic test has not yet been found. The standard fecal occult blood test has a low sensitivity as not all tumours cause bleeding, giving rise to many false negatives. Colonoscopy, though it has a high sensitivity (92–99%) for both pre-malignant lesions and tumours, is invasive, inconvenient for the patients and associated with high-cost^5^, consequently inappropriate as screening tool to identify early stages of CRC. Thus alternative diagnostic tools have to be identified.

Serological biomarkers have the advantages of being easy to collect, non-invasive, typically low-cost, and have the ability to be followed over the course of the disease. Identification of serological biomarkers that can aid in early detection, diagnosis, disease monitoring and in individual treatment selection of CRC patients could have a high impact on the patient outcome.

The local microenvironment of a tumour has been shown to play important roles in tumour pathogenesis and there is much focus on the extracellular matrix (ECM) and its remodeling as contributor to malignancy^6^,^7^. The ECM is constantly being remodeled and in the healthy tissue there is a balanced ratio between degradation and formation of ECM proteins. Disruption of this homeostasis may act as a driver of cancer development and invasion. Excessive ECM remodeling is characterized both by an increased collagen deposition (desmoplasia) and crosslinking leading to tissue stiffening as well as an increased expression of matrix metalloproteinases (MMPs)^6^,^8^. As a consequence, increased levels of tissue - and cancer-specific ECM turnover products, so-called neo-epitopes, are released into the circulation making them potential as novel blood-based biomarkers. Many of these proteins have been uniquely modified by the pathology, such as the generation of a unique degradation site by a cancer dependent protease (e.g. MMP) in a signature protein (e.g. collagen) providing a so-called protein fingerprint. This combination is more related to pathogenesis than unmodified proteins and therefore potential candidates for novel biomarkers in cancer^9^.

Neo-epitope biomarkers have been shown to have a diagnostic potential in lung cancer^10^, breast cancer^11^, ovarian cancer^11^ and pancreatic cancer^12^. In this study, we investigated whether serum biomarkers reflecting collagen remodeling could differentiate between colorectal cancer patients, subjects with adenomas and healthy controls. Furthermore, we examined if the individual biomarkers or combinations hereof could be used to stratify patients according to their tumour stage.

## Results

### Collagen remodeling biomarkers are elevated in colorectal cancer patients compared to subjects with adenomas and healthy controls

**Figure 1:**
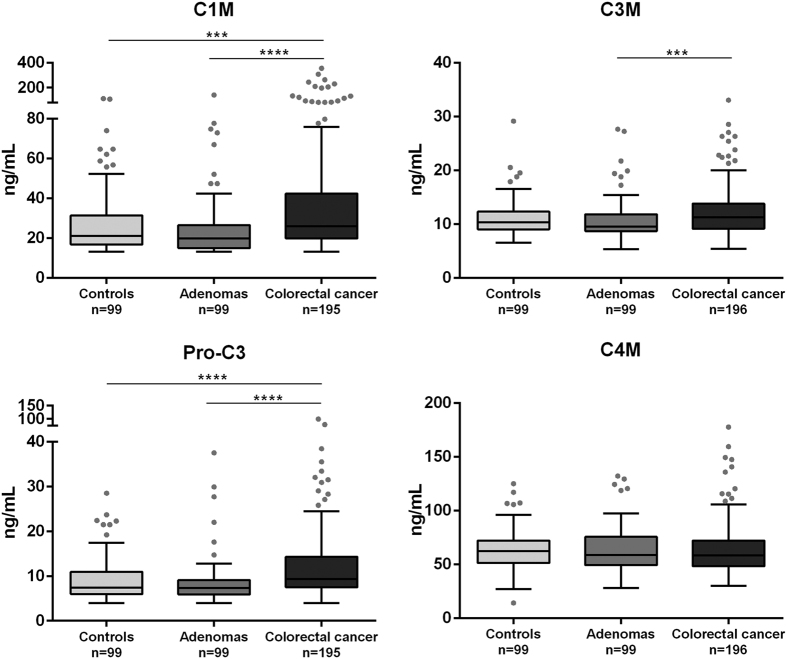
Serum levels of MMP-mediated degradation of type I collagen (C1M), type III collagen (C3M) and type IV collagen (C4M) and formation of type III collagen (Pro-C3). Groups were compared using a Kruskal–Wallis test. The p-values were adjusted to account for multiple comparisons using Dunnett’s method. Significance levels: **p < 0.01, ***p < 0.001 and ****p < 0.0001.

Serum levels of biomarkers specifically reflecting type I (C1M), type III (C3M) and type IV (C4M) collagen degradation and type III collagen formation (Pro-C3) were measured in healthy controls, subjects with adenomas and colorectal cancer patients. The data are presented in Fig. 1. In detail, the level of C1M and Pro-C3 were significantly elevated in colorectal cancer patients compared to healthy controls (C1M: p < 0.001, Pro-C3: p < 0.0001) and subjects with adenomas (C1M: p <0.0001, Pro-C3: p <0.0001). C3M was significantly elevated in colorectal cancer patients compared to subjects with adenomas (p < 0.001). No significant difference was observed between the groups for C4M.

These data suggest that altered type I and type III collagen remodeling is ongoing in colorectal cancer and not in the healthy tissue and pre-malignant stages. This reflects that an altered collagen turnover and the release of collagen protein fragments to the circulation is a pathological feature of colorectal cancer.

### Collagen remodeling biomarkers are elevated in stage IV colorectal cancer patients

**Figure 2:**
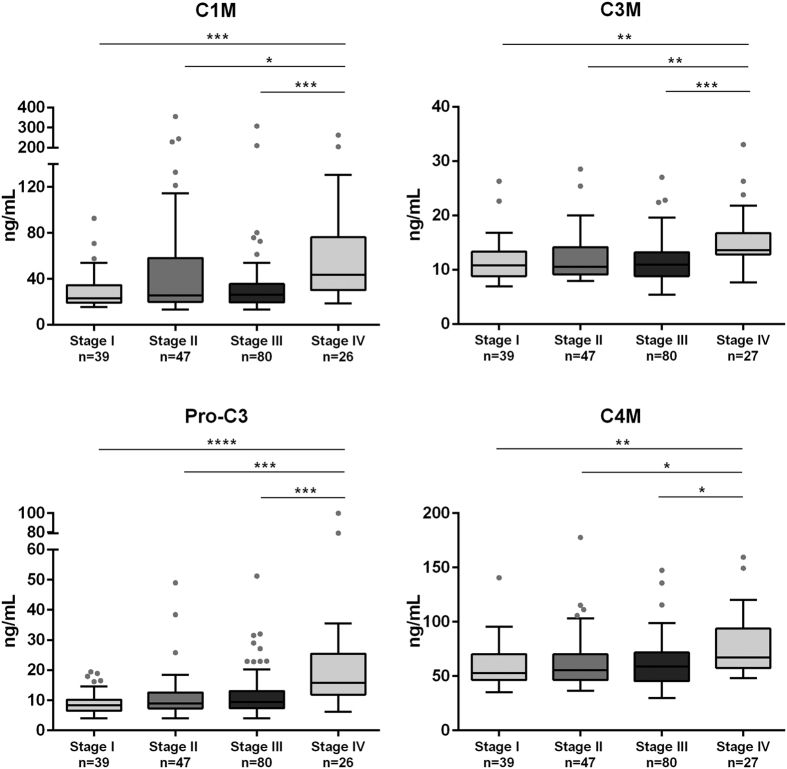
Serum levels of MMP-mediated degradation of type I collagen (C1M), type III collagen (C3M) and type IV collagen (C4M) and formation of type III collagen (Pro-C3) in colorectal cancer patients stratified according to their tumour stage. Groups were compared using a Kruskal–Wallis test. The p-values were adjusted to account for multiple comparisons using Dunnett´s method. Significance levels: *p < 0.05, **p < 0.01, ***p < 0.001 and ****p < 0.0001.

To investigate if the collagen remodeling biomarkers could be used to stratify patients according to their tumour stage, we divided the colorectal cancer patients into four groups according to their tumour stage, excluding the three patients with carcinoma *in situ* (Table 1). The data are presented in Fig. 2. All four biomarkers were significantly elevated in patients with stage IV tumours compared to all other stages (p < 0.05 to <0.0001, dependent on the marker). No differences were observed among patients diagnosed with stage I-III. The high level of all markers in stage IV patients indicates that collagen remodeling takes part in colorectal cancer progression and invasion and that these four biomarkers are a measure of tumour activity in advanced stages.

### Discriminative power of collagen remodeling biomarkers for metastatic and non-metastatic colorectal cancer

The area under the receiver operating characteristics (AUROC) and logistic regression was used to evaluate the discriminative power of the four biomarkers for metastatic and non-metastatic colorectal cancer (Table 2). As shown in Table 2, Pro-C3 and C3M displayed the highest diagnostic accuracy in relation to identify metastatic tumours from non-metastatic tumours with an AUROC of 0.79 and 0.78, respectively. For each biomarker, the optimal cut-off value was determined by ROC curve analyses and the sensitivity and specificity are shown in Table 2.

We performed logistic regression analyses to calculate the best diagnostic value by combining all biomarkers and to correct for confounding factors (age and gender). Tumour stage (non-metastatic vs metastatic (stage IV)) was included as dependent variable and the four biomarkers, age and gender as explanatory variables. In this logistic regression model, C3M and Pro-C3 were found to be statistically significant (C3M: p = 0.006, Pro-C3: p = 0.002) and the diagnostic value for separating metastatic patients from non-metastatic patients increased to an AUROC of 0.80 (95% CI: 0.65–0.91), a sensitivity of 92.6% and a specificity of 67.3% when 0.097 was used as cut-off value. Based on the logistic regression model, we propose the following algorithm which could be used as an aid for clinicians to manage patients that have been diagnosed with colorectal cancer and to assess the severity and tumour progression/activity by collecting a blood sample and measure Pro-C3 and C3M concentrations in serum: Tumour activity score = −4.96 + 0.16 × C3M + 0.07 × Pro-C3. Applying this model to the data from the current study, an AUC of 0.83 is achieved for detecting advanced stage colorectal cancer patients. This model/algorithm needs to be further validated in large clinical studies in order to identify the appropriate score for advanced stage colorectal cancer.

## Discussion

In the present study we measured a panel of four collagen remodeling biomarkers in serum from patients with colorectal cancer, subjects with adenomas and healthy controls. To our knowledge, this is the first study investigating the diagnostic potential of type I, III and IV collagen turnover biomarkers in colorectal cancer. The main finding was that type I, III and IV collagen remodeling biomarkers were significantly elevated in stage IV metastatic colorectal cancer compared to all other stages. Combination of all four biomarkers resulted in differentiation between metastatic and non-metastatic patients with an AUROC of 0.80, while Pro-C3 alone gave rise to an AUROC of 0.79. Together, the data demonstrate that these biomarkers, especially Pro-C3, can be used to assess tumour activity/invasiveness in patients diagnosed with colorectal cancer based on a blood sample. Moreover, the data indicate that the excessive collagen degradation (C1M, C3M and C4M) and formation (Pro-C3) observed in metastatic colorectal cancer patients could play a role in cancer pathogenesis, either as a driver of tumour progression or by being a consequence hereof.

Elevated levels of collagen-derived fragments have been detected in other cancer types, including lung-10, breast-11, ovary-11 and pancreatic cancer^12^. Our results together with these data support that ECM remodeling is an important part of and/or contributes to cancer development and progression^6^,^7^. In fact, it is becoming widely accepted that ECM remodeling and dysregulation directly affect the hallmarks of cancer as defined by Hanahan and Weinberg in 2000^13^,^14^,^15^.

C1M and C3M reflects interstitial matrix remodeling, where the C1M assay measures a type I collagen degradation fragment generated by cleavage with MMP-2, −9 and −13^16^ and the C3M assay measures a type III collagen degradation fragment generated by cleavage with MMP-9^17^, respectively. C4M reflects basement membrane remodeling and measures a type IV collagen α1- chain fragment generated by cleavage with MMP-12^18^. These four MMPs (2, 9, 12 and 13) have in fact been associated with colorectal cancer supporting an increased collagen degradation^19^,^20^,^21^. The disruption of the basement membrane and the interstitial matrix is an essential prerequisite for tumour invasion. For tumour cells to metastasize, they must not only degrade the matrices of the colon wall, but also the matrices of lymphatic system, blood vessels and at the secondary site. Degradation of the interstitial matrix paves the way for the tumour cells thereby enhancing migration across the matrix to nearby lymph nodes and blood vessels^19^,^22^. In the current study, C1M, C3M and C4M were significantly elevated in stage IV patients. In theory, we would expect the basement membrane and interstitial matrix of the colon wall to be degraded at early stages of invasion (stage I-III), however this was not detectable in serum by the current biomarker assays. This may be explained by a lower MMP activity at earlier tumour stages or perhaps by increased cellular uptake of collagen fragments in initial stages. Our results might rely on an ongoing degradation of both the basement membrane and interstitial matrix of the colon wall leading to tumour progression into deeper tissues and excessive release of collagen turnover products into the circulation as a consequence of metastasis. At this stage, the basement membrane and interstitial matrix of the lymphatic system, blood vessels and the secondary site might also have been breached, likewise resulting in enhanced levels of collagen fragments detectable in serum.

Pro-C3 measures the true formation of type III collagen as the antibody is directed against the cleavage site of the N-terminal collagen III pro-peptide^23^ which are released during collagen formation and maturation. This marker has been extensively studied in liver fibrosis as a biomarker of progression and burden of disease^24^,^25^,^26^,^27^. Cox and Erler^28^ have recently reviewed the link between fibrosis and tumour metastasis. It was discussed that tumour cells can prepare a pre-metastatic niche in a manner that resembles the development of fibrosis. In order to convert an unfavorable tumour environment of a distant site into favorable surroundings, tumour cells secrete a lot of factors before and upon arrival at the metastatic niche. This includes factors that promote increased collagen deposition and crosslinking as observed in fibrotic tissue. The increased formation of type III collagen measured in stage IV patients may originate from the metastatic niche either as a result of tissue priming or upon tumour cell arrival. This is in line with several studies showing the importance of increased collagen deposition in the metastatic niche for further tumour progression^29^,^30^,^31^,^32^.

Identification of neo-epitope biomarkers that directly reflect the changes of the extracellular matrix during cancer could be a new way handling the medical need in colorectal cancer. Each neo-epitope results from a specific pathological process giving rise to a very unique and specific biomarker^9^. The fact that the biomarkers were significantly elevated in stage IV patients and had a high discriminative value for metastatic patients, clearly indicates that the level of collagen remodeling biomarkers is a measure of tumour activity and severity. Therefore, it is likely that these biomarkers could be novel candidates as tools to monitor colorectal cancer patients with advanced stages. Such tools would be valuable since it is often the metastases rather than the primary tumour which cause the poor prognosis of cancer patients^33^. Blocking the invasion of cancer and its growth at the distant site could therefore improve the patient outcome. The ECM is in fact an emerging target for cancer drug therapy^34^,^35^,^36^. Several preclinical studies have investigated key ECM proteins as targets for novel cancer drugs (reviewed in ref. 34). The present biomarkers have a potential as treatment of efficacy biomarkers in relation to ECM modifying drugs. Since the four biomarkers investigated here reflect excessive ECM remodeling, they might be able to identify patients with a densely remodeled stroma who would benefit most from ECM targeting drugs. Furthermore, the biomarkers could be used to monitoring advanced stage patients after therapy, as we hypothesize that circulating levels would decrease after successful treatment of the tumour creating a homeostatic tissue environment and then increase again if there is recurrence. Carcinoembryonic antigen (CEA) is the most widely used serum biomarker in patients with colorectal cancer^37^. The poor sensitivity of this test for early stage tumours makes it unsuitable for screening and early detection and the main use of CEA in colorectal cancer is for surveillance and monitoring before and after surgery/treatment. However the usefulness of CEA is controversial^37^,^38^,^39^. CEA is an oncofetal antigen whereas the investigated biomarkers are designed as a measure of tumour activity, invasiveness and pathology specific which may increase specificity and sensitivity. However, this has to be validated in larger cohorts together with CEA measurements.

One limitation of this study is its cross-sectional nature. To fully elucidate the prognostic applicability and treatment efficacy of the collagen turnover biomarkers, larger longitudinal studies are needed. Another limitation is the absence of a replication/validation cohort which would have supported the findings. However, we find these preliminary results to be important steps towards identifying novel blood-based biomarker tools in colorectal cancer.

In conclusion, colorectal cancer is a field with an urgent need for biomarkers that can aid in diagnosis and prognosis. We have assessed a panel of biomarkers reflecting collagen turnover of the extracellular matrix in patients with colorectal cancer, subjects with adenomas and controls. All markers were significantly elevated in stage IV patients suggesting that excessive collagen turnover takes part in cancer progression and metastasis. As these markers are designed to measure tumour activity, they may increase the understanding of cancer pathology and, if validated in larger clinical studies, provide new clinical tools for patient monitoring and efficacy of treatment.

## Methods

### Patient samples

This study included serum samples from 394 individuals comprising 99 healthy controls, 99 patients with adenomas and 196 patients with colorectal cancer at different stages. For all individuals, 9 ml of blood was collected and centrifuged to separate serum. Then, samples were divided into single-use aliquots and preserved at −80 °C. All controls and patients were recruited at the Bellvitge University Hospital (Barcelona, Spain) and all samples were handled the exact same way. Written informed consent was obtained from all patients and the Ethics Committee of the Bellvitge University Hospital approved the protocol with reference PR073/11. The study was carried out in accordance with ICH-GCP and according to the Declaration of Helsinki. Table 1 shows the detailed characteristics of healthy controls, adenomas and colorectal cancer patients.

### Protein Fingerprint biomarker analysis

The Protein Fingerprint biomarkers of matrix metalloproteinase (MMP) degraded type I, type III and type IV collagen (C1M, C3M, C4M) and type III collagen formation (Pro-C3) were assessed in serum as previously described^16^,^17^,^18^,^23^. Briefly, 96-well pre-coated streptavidin plates were coated with biotinylated synthetic peptides specific for the protein of interest and incubated for 30 minutes at 20 °C. 20 μL of standard peptide or pre-diluted serum sample were added to designated wells followed by the addition of peroxidase-conjugated specific monoclonal antibodies and incubated for 1 h at 20 °C or overnight at 4 °C. Finally, tetramethylbenzinidine (TMB) (cat. 438OH, Kem-En-Tec Diagnostics, Denmark) was added to each well and the plates were incubated for 15 minutes at 20 °C. All incubation steps included shaking at 300 rpm and after each incubation step, the plates were washed five times with wash buffer (20 mM Tris, 50 mM NaCl, pH 7.2). The enzymatic reaction was stopped by adding 0.18 M H_2_SO_4_ and absorbance was measured at 450 nm with 650 nm as reference. A calibration curve was plotted using a 4-parameter logistic curve fit.

### Statistical analysis

The levels of the individual biomarkers in serum samples in each group (controls, adenomas and cases) were compared using a Kruskal–Wallis test (non-parametric test). The p-values were adjusted to account for multiple comparisons using Dunnett´s method.

The diagnostic power of individual and combined markers was investigated by the area under the receiver operating characteristics (AUROC). Sensitivity and specificity were determined for appropriate cut-off values based on the ROC curves. Logistic regression analyses were carried out to calculate the best diagnostic value when combining all biomarkers and correcting for age and gender. To correct for over fitting, an internal validation was conducted by calculating the bootstrap optimism-corrected AUC (the data were resampled 1000 times with the bootstrapping method).

Unless otherwise stated, data are shown as Tukey box plots, where the boxes represent the 25th, 50th and 75th percentiles. The whiskers represent the lowest and highest value, except outliers, which are higher than 1.5 times the 75th percentile or lower than 1.5 times the 25th percentile. P-values <0.05 were considered significant. Statistical analyses were performed using the R statistical computing software (http://www.r-project.org), MedCalc Statistical Software version 12 (MedCalc Software, Ostend, Belgium) and GraphPad Prism version 6 (GraphPad Software, Inc., CA, USA). Graphs were designed using GraphPad Prism version 6 (GraphPad Software, Inc., CA, USA).

## Additional Information

**How to cite this article**: Kehlet, S. N. *et al*. Excessive collagen turnover products are released during colorectal cancer progression and elevated in serum from metastatic colorectal cancer patients. *Sci. Rep.* **6**, 30599; doi: 10.1038/srep30599 (2016).

## Figures and Tables

**Table 1:** Patients demographics and clinical profiles.

Group	No. of subjects	Gender, % females	Age (years) mean ± SD	Carcinoma *in situ* (n)	Tumour stage I (n)	Tumour stage II (n)	Tumour stage III (n)	Tumour stage IV (n)
Healthy controls	99	64.6	60.1 ± 6.3	–	–	–	–	–
Adenomas	99	26.3	59.8 ± 5.9	–	–	–	–	–
Colorectal cancer cases	196	32.1	63.1 ± 10.4	3	39	47	80	27

**Table 2:** Discriminative performance of collagen remodeling biomarkers for detecting metastatic colorectal cancer (stage IV) versus non-metastatic (stage I–III).

Biomarker	Cut-off value (ng/mL)	Sensitivity	Specificity	AUROC (95% CI)	p-value
C1M	30.7	77.8	67.9	0.74 (0.62–0.87)	<0.0001
C3M	11.9	85.7	65.5	0.78 (0.66–0.89)	<0.0001
C4M	52.3	96.4	43.0	0.74 (0.61–0.87)	<0.0001
Pro-C3	10.4	88.9	66.1	0.79 (0.65–0.91)	<0.0001
